# Impact of national guidelines on use of *BRCA1/2* germline testing, risk management advice given to women with pathogenic *BRCA1/2* variants and uptake of advice

**DOI:** 10.1186/s13053-021-00180-3

**Published:** 2021-04-09

**Authors:** Bettina Meiser, Rajneesh Kaur, April Morrow, Michelle Peate, W. K. Tim Wong, Emily McPike, Elisa Cops, Cassandra Nichols, Rachel Austin, Miriam Fine, Letitia Thrupp, Robyn Ward, Finlay Macrae, Janet E. Hiller, Alison H. Trainer, Gillian Mitchell, for the ICCon Audit Study Collaborative Group, R. Susman, N. Pachter, A. Goodwin, P. James, L. Mascarenhas, C. Morton, S. Shanley, M. A. Young, L. Andrews, E. A. Morrow, K. Tucker, P. James, G. Lindeman, L. Mascarenhas, C. Morton, M. Field, A. Goodwin, M. Monnik, N. Poplawski, M. Delatycki, T. John, M. Harris, R. Kerr, B. Vora

**Affiliations:** 1 Psychosocial Research Group, Prince of Wales Clinical School, Faculty of Medicine, UNSW Sydney, Sydney, NSW 2052 Australia; 2 Department of Obstetrics and Oncology, Royal Women’s Hospital, University of Melbourne, Melbourne, Australia; 3 School of Social Sciences, UNSW Sydney, Sydney, Australia; 4 Parkville Familial Cancer Centre, Peter MacCallum Cancer Centre and Royal Melbourne Hospital, Melbourne, Australia; 5 Genetic Services of Western Australia, King Edward Memorial Hospital, Perth, Australia; 6 Genetic Health Queensland, Royal Brisbane and Women’s Hospital, Herston, Queensland Australia; 7 Adult Genetics Unit, Royal Adelaide Hospital, Adelaide and School of Medicine, University of Adelaide, Adelaide, Australia; 8 University of Queensland, Brisbane, Australia; 9 University of Sydney, Faculty of Medicine and Health, Sydney, Australia; 10 Department of Medicine, Royal Melbourne Hospital, Melbourne, Australia; 11 Department of Colorectal Medicine and Genetics, University of Melbourne, Melbourne, Australia; 12 Swinburne University of Technology, Hawthorn, Australia; 13 School of Public Health, Faculty of Health and Medical Sciences, University of Adelaide, Adelaide, Australia; 14 The Sir Peter MacCallum Department of Oncology, University of Melbourne, Melbourne, Australia

**Keywords:** Guidelines, Compliance, Genetic testing, Cancer risk, *BRCA1* and *BRCA2*

## Abstract

**Background:**

This nationwide study assessed the impact of nationally agreed cancer genetics guidelines on use of *BRCA1/2* germline testing, risk management advice given by health professionals to women with pathogenic *BRCA1/2* variants and uptake of such advice by patients.

**Methods:**

Clinic files of 883 women who had initial proband screens for *BRCA1/2* pathogenic variants at 12 familial cancer clinics between July 2008–July 2009 (i.e. before guideline release), July 2010–July 2011 and July 2012–July 2013 (both after guideline release) were audited to determine reason given for genetic testing. Separately, the clinic files of 599 female carriers without a personal history of breast/ovarian cancer who underwent *BRCA1/2* predictive genetic testing and received their results pre- and post-guideline were audited to ascertain the risk management advice given by health professionals. Carriers included in this audit were invited to participate in a telephone interview to assess uptake of advice, and 329 agreed to participate.

**Results:**

There were no significant changes in the percentages of tested patients meeting at least one published indication for genetic testing - 79, 77 and 78% of files met criteria before guideline, and two-, and four-years post-guideline, respectively (χ = 0.25, *p* = 0.88). Rates of documentation of post-test risk management advice as per guidelines increased significantly from pre- to post-guideline for 6/9 risk management strategies. The strategies with the highest compliance amongst carriers or awareness post-release of guidelines were annual magnetic resonance imaging plus mammography in women 30–50 years (97%) and annual mammography in women > 50 years (92%). Of women aged over 40 years, 41% had a risk-reducing bilateral mastectomy. Amongst women aged > 40 years, 75% had a risk-reducing salpingo-oophorectomy. Amongst women who had not had a risk-reducing bilateral mastectomy, only 6% took risk-reducing medication. Fear of side-effects was cited as the main reasons for not taking these medicines by 73% of women.

**Conclusions:**

Guidelines did not change the percentages of tested patients meeting genetic testing criteria but improved documentation of risk management advice by health professionals. Effective approaches to enhance compliance with guidelines are needed to improve risk management and quality of care.

**Supplementary Information:**

The online version contains supplementary material available at 10.1186/s13053-021-00180-3.

## Introduction

Genetic testing in Australia for pathogenic variants in *BRCA1/2* has evolved since the mid-1990s. Although testing has improved and costs have reduced, it is still important to offer genetic testing as a priority to families where it is most likely to be useful. The identification of a causative pathogenic variant allows for tailored management of the cancer-affected individual and for predictive genetic testing for others in the family. Initially, genetic testing was funded by state health departments, and individual family cancer clinics (FCCs) determined the criteria used locally to guide the offer of a *BRCA1/2* genetic test to women affected by breast and/or ovarian cancer. Research generally guided the development of criteria to be used by FCCs for an offer of testing, whereby individuals with at least a 10% chance of having a *BRCA1/2* pathogenic variant were offered testing. However, the Cancer Institute New South Wales convened a national expert group to develop consensus guidelines on criteria for consideration of *BRCA1/2* genetic testing, so that access to such testing would be equitable across Australia. Guidelines were to be based on evidence and/or expert opinion in the absence of evidence. The resulting agreed guidelines were then published and made available on web-based national clinical point-of-care guidelines [1], called eviQ [2]. EviQ guidelines also cover risk management for carriers of pathogenic variants and are reviewed regularly and have been expanded to other hereditary cancer syndromes. Clinicians representing all public FCCs in Australia are involved in guideline development, and therefore notification and dissemination of new or updated guidelines is immediately accomplished at all FCCs through their contributing clinicians. The extent to which clinician advice on risk management according to cancer genetics guidelines translates into patients taking up this advice may be affected by many factors including patient preference and patients’ ability to access risk management. Access is affected by geography, local facilities (medical and investigational), and patient financial and social resources.

While rigorous controlled trials of cancer risk management strategies are few in the hereditary breast/ovarian cancer field, there is increasing evidence for the use of specific cancer risk management interventions, such as preventing ovarian cancer and reducing the risk of breast cancer associated with *BRCA1/2* pathogenic variants [3]. Individuals at a high risk of developing cancer, due to an inherited cancer syndrome, provide an ideal focus for screening and medical/surgical prevention strategies due to the attractive benefit/risk ratio.

Female carriers of pathogenic variants in *BRCA1/2* have a high lifetime risk of breast and ovarian cancer. For carriers of pathogenic variants in *BRCA1*, the average cumulative risks by age 80 years is 72% (95% confidence interval 65–79%) for breast cancer and 44% (36–53%) for ovarian cancer, and the corresponding estimates for *BRCA2* are 69% (61–77%) and 17% (11–25%) respectively [4]. Carriers of pathogenic variants in *BRCA1/2* may consider risk-reducing bilateral mastectomy to reduce cancer incidence by up to 95% and breast cancer-specific mortality by 80% [5–7]. Carriers may also opt for risk-reducing bilateral salpingo-oophorectomy around age 40 years (after childbearing is complete). This reduces the risk of ovarian cancer by up to 95% in carriers of pathogenic variants [8], may reduce the risk of breast cancer *(BRCA2)* [9, 10] and reduces breast/ovarian/all-cause mortality [11]. Women at increased risk of breast cancer, including women with a *BRCA1/2* pathogenic variant, may also consider use of selective oestrogen receptor modulators (e.g. tamoxifen) as a risk-reducing strategy. Studies have shown that the use of such agents can reduce the risk of developing oestrogen-receptor-positive breast cancer by up to 50% [12]. Finally, most guidelines relating to carriers of pathogenic *BRCA1/2* variants recommend annual magnetic resonance imaging (MRI) in combination with mammography and/or ultrasound as a screening tool to maximise the sensitivity of breast cancer screening in this group [2, 13–16].

However, simply having guidelines agreed by national experts and published is not sufficient; in order to improve clinical outcomes the appropriate advice (adjusted for age, health status and personal preference) must be provided to each woman and to her primary health professional. Clinical practice guidelines aim to facilitate decision-making processes in patient care and have been shown to be capable of supporting improvements in quality and consistency in healthcare [17]. However, merely disseminating guidelines is usually not sufficient to affect clinical practice [18], and therefore evidence is required to assess the degree to which guidelines are translated into clinical practice [19]. Almost no literature is currently available on the impact of cancer genetics clinical practice guidelines. However, we recently reported on the impact of guidelines on risk management for carriers of pathogenic variants in mismatch repair genes [20]. We found that the rates of documentation of risk management advice in clinic files increased significantly from pre- to post-guideline publication for only two out of eight risk management strategies [20], and the question arises whether risk management advice is more consistently documented for other familial cancer syndromes. In our previous study, we also found high compliance of carriers post-guideline of risk management strategies, including colonoscopy (87% compliance) [20]. Similar data are needed for female carriers of *BRCA1/2* pathogenic variants.

This current study addresses this gap in the literature by investigating two key areas. The first part of the study investigated the impact of the publication of Australian clinical practice guidelines on use of *BRCA1/2* germline testing for women with breast and/or ovarian cancer. The study also assessed documented compliance by clinicians with respect to national risk management guidelines for unaffected female *BRCA1/2* pathogenic variant carriers. Furthermore, the study surveyed patients to investigate their uptake of strategies from the national risk management guidelines. Specifically, the study aimed to assess the extent to which carriers adhered to recommended risk management guidelines. Finally, the patient surveys permitted exploratory questions about perceived barriers or reasons underlying any failure to undertake recommended risk management strategies.

## Methodology

The design of the study was an uncontrolled before and after design. Such a design is considered appropriate for guideline evaluation, because randomised controlled trials are not feasible for practical and ethical reasons [19].

Australia has 18 public familial cancer clinics (FCCs); their functions vary in relation to coordinating and delivering care to carriers of pathogenic *BRCA1/2* variants. FCCs without a risk management service refer patients back to their non-genetics medical specialist or primary care physician for ongoing care, while those with an associated risk management service manage at least some of the patients themselves. Risk management advice at FCCs is provided by clinicians with medical qualifications (clinical geneticists, breast/gynaecological cancer specialists or oncologists with expertise in familial cancer) as well as genetic counsellors, who are not medically trained. For this study, participation occurred at 12 out 18 of public FCCs (four in Victoria, five in New South Wales, and one each in South Australia, Western Australia and Queensland) following approval from the head of each of the FCCs, who was invited to participate via an invitation email that described the study.

### File audits

Two file audits were undertaken at each FCC to (i) assess eligibility for *BRCA1/2* germline testing against eviQ cancer genetics guidelines amongst women who were tested and (ii) document risk management advice given to unaffected female *BRCA1/2* pathogenic variant carriers. The criteria for eligibility for genetic testing and the risk management recommendations for carriers are presented in Tables 1 and 2 respectively.

**Table 1:** Number and percentage of files meeting genetic testing reasons (*n* = 883)

Genetic testing reason	Meets reason pre-guideline ***n*** = 280 n (%)	Meets reason two-years post-guideline ***n*** = 301 n (%)	Meets reason four-years post-guideline ***n*** = 302 n (%)
Manchester score ≥ 16^a*^	170/233 (73)	152/242 (63)	151/249 (61)
BOADICEA score ≥ 10^b^	6/7 (86)	7/13 (54)	10/23 (44)
BRCAPRO score ≥ 10^b^	33/48 (69)	37/57 (65)	27/52 (52)
Individuals with triple negative breast cancer, (age ≤ 40 yrs)*	72/280 (26)	22/300 (7)	24/301 (8)
Individuals with isolated high-grade (Grades 2 & 3) invasive, non-mucinous ovarian, fallopian tube or primary peritoneal cancer (age ≤ 70 yrs)*	11/280 (4)	13/300 (4)	20/302 (7)
Individual with high-grade serous or endometroid (or invasive non-mucinous) ovarian, fallopian tube or primary peritoneal cancer and a family history*	9/280 (3)	19/301 (6)	36/302 (12)
Met at least one criterion	192/243 (79)	217/281 (77)	220/281 (78)

Women who self-funded testing were excluded. ^*^Denominators vary due to missing data. ^a^Manchester scores for each woman relating to a file were calculated by ICCon co-ordinators rather than extracted from files. ^b^Denominator relates to the number of files where score is mentioned

**Table 2:** Number and percentages of reasons given for non-compliant files^a^

Reasons given	Pre-guideline ***n*** = 85 n (%)	2-years post-guideline ***n*** = 94 n (%)	4-years post-guideline ***n*** = 90 n (%)	Total ***n*** = 269 n (%)
Enrolled in the Treatment Focused Genetic Testing Study^b^	0 (0)	3 (3)	0 (0)	3 (1)
Gender imbalance-pedigree includes mostly males	3 (3)	2 (2)	5 (6)	10 (4)
Small family size-not enough people in the pedigree to allow an estimation of prevalence	2 (2)	7 (7)	4 (4)	13 (5)
There is ovarian cancer in the family	10 (12)	5 (5)	1 (1)	16 (6)
Patient was adopted	3 (3)	1 (1)	1 (1)	5 (2)
Patient did not know about their family history	1 (1)	0 (0)	0 (0)	1 (0)
Patient’s breast cancer diagnosis < 40 years	15 (18)	22 (23)	28 (31)	65 (24)
Other	51 (60)	54 (57)	46 (51)	151 (56)

^a^More than one reason could be mentioned per test ordered. Women who self-funded testing were excluded

^b^Trial offering genetic testing for affected women aged < 50 years with high-risk features

^c^Count based on responses to open-ended item “Other reasons” in Supplementary File 1

### File audit to investigate use of *BRCA1/2* genetic testing

For the file audit to ascertain women’s eligibility for genetic testing for *BRCA1/2,* the Queensland FCC did not participate, because they used a higher minimum positive predictive value to identify a *BRCA1/2* pathogenic variant (20%) compared to other FCCs (10%) to determine eligibility for *BRCA1/2* testing at the time of the study. For the audit on reasons for genetic testing, participating FCCs prepared a list of all women who had genetic testing during three time periods: July 2008–July 2009 (i.e. before guideline release), July-2010-July 2011 and July 2012–July 2013 (both after guideline release). Women who self-funded *BRCA1/2* genetic testing were excluded, because genetic testing eligibility guidelines do not apply to this group. From these lists, up to 32 women per FCC and per assessment time period were randomly selected. Due to the roll out of the guidelines at slightly different times at different clinics, the three assessment time periods were separated by 12-months periods.

A checklist was completed by the local ICCon co-ordinator to indicate the reason the woman relating to the file was offered genetic testing. An example checklist is provided in Supplementary File 1. Two slightly revised versions of genetic testing guidelines were published during the assessment period: Version 1 (June 2010–February 2011) and Version 2 (October 2011–July 2013), although there were minimal differences, and appropriate checklists corresponding to each version were used. The main differences were that Version 2 of the genetic testing guideline had: (i) a Manchester score [21] cut-off of 16 instead of 15, and that (ii) the term “serous or endometroid” was replaced with “invasive non-mucinous”. If non-compliant, the ICCon co-ordinator completing the checklist extracted reasons for non-compliance from clinic files (see Supplementary File 1, Point 2.f, for a list of potential reasons).

### File audit on impact of risk management guidelines

As an initiative of the Inherited Cancer Connect (ICCon) Partnership, a national database has been established to house de-identified data on individuals found to carry a pathogenic variant in a known hereditary cancer susceptibility gene through all public Australian FCCs. Using ICCon data, study co-ordinators working in the participating FCCs prepared lists of all female carriers of pathogenic variants in *BRCA1/2* genes without breast/ovarian cancer who had undergone predictive testing and were identified as carriers during the three assessment time periods. We were unable to include enough carriers for the third time period for the guidelines on risk management, and hence the files from this period were not included in the analyses on risk management.

Similar to the genetic testing eligibility audit, a checklist for each guideline was completed by the local ICCon co-ordinator to indicate whether the file related to each of the included carriers documented that the woman was informed about each risk management strategy listed in the guideline. An example checklist for the risk management file audit is provided in Supplementary File 2. A file was deemed concordant with guidelines if the information on risk management was documented in the files as having been either provided verbally to the woman during the consultation or through a letter to the referring doctor with a copy to the carrier or via a fact sheet given to individual. Two revised versions of risk management guidelines for *BRCA1* were published: Version 1 (July 2011–June 2012) and Version 2 (July 2012–December 2013). The two versions did not differ on age-specific advice about the main risk management strategies of breast and ovarian cancer screening, surgical prevention and risk-reducing medication; however they were updated for lifestyle advice. Appropriate checklists corresponding to each version were used.

### Interviews with carriers of *BRCA1/2* pathogenic variants

An invitation letter was sent by heads of FCCs to those carriers of *BRCA1/2* pathogenic variants included in the risk management advice file audit to participate in a telephone interview. Participants were given the option of opting out via mail, phone or fax if they preferred not to be contacted. Two weeks after the letters were sent out, the local ICCon co-ordinator called women who had not opted out to investigate their uptake of risk management strategies, using a checklist to record whether or not the carrier had implemented each of the risk management strategies. An example of an interview checklist is shown in Supplementary File 3.

### Data analysis

The checklists were completed electronically using the University of New South Wales survey provider and analysed by the central study co-ordinator using the statistical software SPSS (Statistical Programme for the Social Sciences) Version 25. For the files on guidelines for genetic testing use, a Manchester score [21] was calculated by the local ICCon co-ordinator based on information from the pedigree (if a Manchester score was not mentioned in the file). About 10% of the audited files were randomly selected and independently rated by the central study co-ordinator to calculate inter-rater reliability of compliance ratings. For the file audit, the files relating to women were classified as concordant or non-concordant to guidelines with respect to documented advice for each risk management strategy. Similarly, for the interviews, women were classified as concordant or non-concordant with guidelines with respect to each risk management strategy, based on their age at the time of interview. Given risk management guidelines for *BRCA1* and *BRCA2* pathogenic variant carriers were quite similar, the data on carriers of *BRCA1* and *BRCA2* pathogenic variants were combined for analyses. Chi square and Kruskal-Wallis tests were performed to compare the rates of compliance before and after the introduction of guidelines. Significance level was set at *p* < 0.05. Reasons for non-compliance, which were provided using open-ended response options, were extracted, categorised and then tallied.

## Results

### File audit to determine reasons for *BRCA1/2* genetic testing

Interrater reliability coefficients (k statistics) for assessing individual guidelines were between 0.79–0.87 for reasons for *BRCA1/2* genetic testing, and the overall k statistic was 0.84. A total of 883 files were audited for the genetic testing reason audit over the three time periods. For the 11 participating FCCs, the number of files ranged between 7 and 35 for the pre-guideline period, and between 12 and 35 and between 13 and 35 for the two-years and four-years post-guideline periods respectively.

Table 1 shows the number of files relating to women eligible for *BRCA1/2* testing against set criteria for testing. Before the release of the guidelines, 79% of the files met at least one of the criteria, while 21% of files did not meet any of the criteria. Two- and four-years post-guideline release, 77 and 78% of files met at least one genetic testing criterion respectively. The compliance to genetic testing guidelines was not significantly different between the three time periods (χ = 0.25, *p* = 0.88).

Table 2 summarises the reasons indicated in the files for non-compliance. Table 2 demonstrates that the three most frequent reasons across the three time periods taken together were that the patient was diagnosed with breast cancer under the age of 40 years (24%), there was ovarian cancer in the family (6%) and small family size (5%). No reasons for testing were provided for all but 14 women who did not meet guideline-based testing criteria pre-guideline, while at two- and four-years post-guideline a reason for testing was documented for all women who did not meet guideline-based criteria.

### File-documented recommendations on risk management strategies of carriers

Interrater reliability coefficients (k statistics) for assessing documentation of individual guidelines in patient records were between 0.85–0.94 for risk management of carriers of *BRCA1/2* pathogenic variants and the overall k statistic was 0.92. A total of 599 files were audited, and of these, 316 (53%) and 283 (47%) related to carriers of pathogenic variants in *BRCA1* and *BRCA2* respectively. Of these 373 (62%) files were from the time period before the guidelines were published and 226 (38%) from the post-guideline time period. For the 12 participating FCCs, the number of files ranged between 12 and 58 for the pre-guideline period, and between 6 and 33 and between 4 and 53 for the two- and four-years post-guideline periods respectively.

Results of comparisons of clinicians’ recommendations documented in women’s files on risk management strategies pre- and post-release of guidelines are shown in Table 3. The rates of documentation of guidelines for 6/9 risk management items increased significantly from pre- to post-release of guidelines. These include the items which have the most significant impact on cancer risk and mortality, namely risk-reducing mastectomy and risk-reducing bilateral salpingo-oophorectomy.

**Table 3:** Number and percentages of files concordant pre- and post-release of guidelines with advice on risk management strategies provided to carriers

Risk management strategy	Pre-guideline concordant ***n*** = 373 n (%)	Post-guideline concordant ***n*** = 226 n (%)	Pre- vs post-guideline ***p***-value
**Breast cancer**			
RRBM ≤40 years followed by self-surveillance of chest wall area*	302/365 (83)	206/224 (92)	**0.002**
Alternatively, in the absence of RRBM, has RRSO been discussed, preferably ≤40 years or before menopause to reduce breast cancer risk?*	307/356 (86)	182/218 (84)	0.37
If breast cancer has been diagnosed in this family < 35 years, has screening been recommended from 5 years prior to earliest age affected?^a^	79/128 (62)	42/63 (67)	0.51
30–50 years - annual MRI + MMG (if MRI unavailable, annual MMG + US), consider 6-month interval US +CBE*	257/334 (77)	182/206 (88)	**0.001**
> 50 years - annual MMG+/− US+ CBE*	138/281(49)	85/175 (49)	0.91
If pregnant - no MRI or MMG, consider US	7/65 (11)	10/34 (29)	**0.02**
Risk-reducing medication in women > 35 years (such as tamoxifen or raloxifene)*	141/373 (38)	133/226 (59)	**< 0.001**
**Ovarian/fallopian tube cancer**			
RRSO age ≤ 40 years (if ovaries present)*	301/356 (85)	213/218 (98)	**< 0.001**
Has the patient been told that screening using serum CA125 and/or transvaginal ultrasound (TVU) is not recommended?^*^	202/359 (56)	167/222 (75)	**< 0.001**

^*^Denominators vary due to missing data. Legend: *RRBM* Risk-reducing bilateral mastectomy, *RRSO* Risk-reducing salpingo-oophorectomy, *MRI* Magnetic resonance screening, *MMG* Mammography, *US* Ultrasound, *CBE* Clinical breast examination, *HRT* Hormone replacement therapy. ^a^Only includes women with a family history below age 35 years

Overall documentation of advice around items of risk management strategies following guideline release ranged from 29 to 98%. The highest rates of documentation post-release were for the following recommended strategies: risk-reducing salpingo-oophorectomy for ovarian cancer prevention (98%), risk-reducing bilateral mastectomy (92%), and breast cancer screening in 30- to 50-year-old women (88%). Low rates of documentation post-release were annual screening in women above 50 years of age (49%); however, 50 and 67% of pre- and post-guidelines files respectively stated that the patient was still young and that it would be discussed at an appropriate age. Additional analyses showed that amongst women aged > 50 years, only 30% were documented as being provided with advice on annual mammography (+/− ultrasound + clinical breast examination). Also, the lowest rate of documentation was found for: “If pregnant - no MRI or mammography and consider ultrasound if pregnant” (29%). However, 62 and 67% of pre- and post-guidelines files respectively stated that management of screening during pregnancy was left to the specialist.

**Fig. 1:**
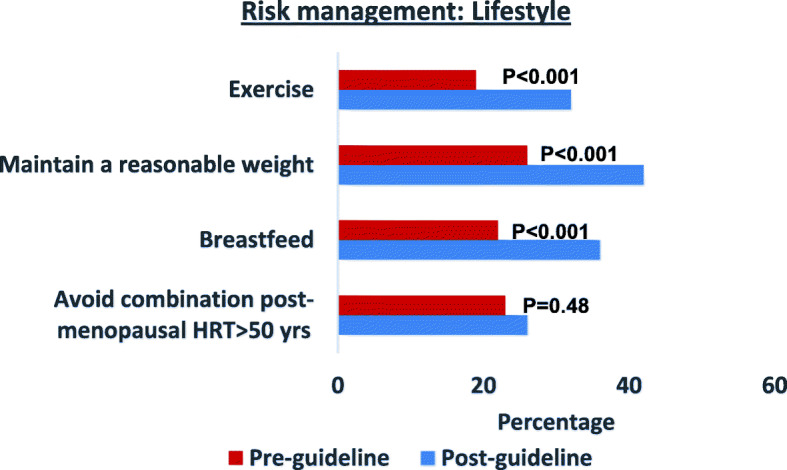
Risk management - Comparison of documented lifestyle-related advice for time periods before and after release of guidelines

Figure 1 shows significant increases from pre- to post-release of guidelines in documentation of advice on the following lifestyle factors: exercise (χ^2^ = 13.96, *p* < 0.001), maintaining a reasonable weight (χ^2^ = 17.83, *p* < 0.001) and breastfeeding (χ^2^ = 20.43, *p* < 0.001). No significant changes were observed in documentation of advice on avoiding post-menopausal HRT after 50 years of age (χ^2^ = 1.57, *p* = 0.48). Despite the increase of the documentation of lifestyle-related advice, it was below 50% for all lifestyle factors (Fig. 1) post-guidelines.

### Carriers’ uptake of risk management strategies

Of the 599 eligible carriers of pathogenic variants without a personal history of breast/ovarian cancer invited to the study, 313 participated in interviews (participation rate of 52%). The mean age of the women at the time of interview was 40 years old (SD = 13.1, range 21 to 90). Table 4 shows the number and percentages of carriers concordant with each risk management strategy. For each risk management recommendation, only women who should have implemented the recommendation based on age are included as the denominator. For example, for risk-reducing bilateral salpingo-oophorectomy only women aged 40 years and over at survey completion who had undergone the risk management strategy are shown, given that women are recommended to complete the strategy by age 40. The strategies with the highest compliance amongst carriers or awareness post-release of guidelines were annual MRI + mammography in women 30–50 years (97%) and annual mammography in women > 50 years (92%). Of women aged over 40 years, only 41% had a risk-reducing bilateral mastectomy. Amongst women aged > 40 years, 75% had a risk-reducing salpingo-oophorectomy. Amongst women who had not had a risk-reducing bilateral mastectomy, only 6% took risk-reducing medication; all of these took tamoxifen, except one woman who took anastrozole. Only 58% of women reported having been told that serum CA125 and/or transvaginal ultrasound is not recommended as a screening test for ovarian cancer.

**Table 4:** Number and percentages of carriers concordant with risk management strategies based on interviews (*n* = 313)

Risk management strategy	Carriers concordant ***n*** = 313 n (%)
RRBM ≤40 years^a^	70/169 (41)
RRSO ≤40 years^a^	127/169 (75)
30–50 years - annual MRI + MMG (if MRI unavailable, annual MMG + US), consider 6-month interval US +CBE^b^	92/95 (97)
> 50 years - annual MMG+/− US+ CBE^c^	46/50 (92)
If pregnant - no MRI or MMG, consider US^d^	14 /18 (78)
Risk-reducing medication in women (such as tamoxifen or raloxifene)^e^	6/100 (6)
Has the patient been told that serum CA125 and/or transvaginal ultrasound (TVU) is not recommended?	176/301 (58)

Legend: *RRBM* Risk-reducing bilateral mastectomy, *RRSO* Risk-reducing salpingo-oophorectomy, *MRI* Magnetic resonance screening, *MMG* Mammography, *US* Ultrasound, *CBE* Clinical breast examination, *HRT* Hormone replacement therapy. ^a^Includes only women aged > 40 years as the denominator. ^b^Includes only women aged 30 to 50 who have not had RRBM. ^c^Includes only women > 50 years who have not had RRBM. ^d^Includes only pregnant women who have not had RRBM. ^e^Includes only women who have not had RRBM

### Reasons for non-compliance regarding risk management strategies

#### Risk-reducing bilateral mastectomy

Amongst women aged < 40 years, 26% had a risk-reducing bilateral mastectomy, amongst those aged 40–59 years 49%, and amongst those aged 60+ years 29%. The most common reasons cited for not having had a risk-reducing bilateral mastectomy were (all ages): young age (53%); family not completed yet (44%); pregnancy or breastfeeding (9%); or being happy to have regular screening (60%). (Note: more than one reason could be given).

#### Risk-reducing salpingo-oophorectomy

Twenty-three percent of women aged < 40 years, 71% of those aged 40–59 years, and 73% of women aged 60+ years, had a risk-reducing salpingo-oophorectomy. The most common reasons cited for not having risk-reducing salpingo-oophorectomy were young age (53%) and still completing family (44%).

#### Risk-reducing medication

As we expected few of the women interviewed use risk-reducing medication, we pre-planned to ask all women participating in interviews who had not had risk-reducing bilateral mastectomy: “To what extent do you agree that each of the following influences your decision whether or not to use a medicine to reduce your risk of developing breast cancer?” Attitudes were assessed with Likert-type response options. Responses are shown in Table 5.

**Table 5:** Attitudes to risk-reducing medication^a^

Statement	Very much agree n (%)	Somewhat agree n (%)	Somewhat disagree n (%)	Very much disagree n (%)
The side-effects of these medicines may affect my decision to take medicine (*n* = 236)	133 (56)	50 (21)	20 (8)	33 (14)
Other people’s experiences with it (*n* = 224)	68 (30)	60 (27)	45 (21)	51 (23)
That is also used as a cancer medicine (*n* = 224)	37 (17)	41 (18)	62 (28)	84 (38)
It is a reminder of your breast cancer risk (*n* = 227)	25 (11)	23 (10)	44 (19)	135 (60)

^a^Includes only women who have not had a risk-reducing mastectomy

Seventy-seven percent women agreed ‘somewhat’ or ‘very much’ that the most common reasons affecting their decision to not take risk-reducing medication was fear of side-effects, and 57% ‘somewhat’ or ‘very much’ agreed that it was other women’s experiences with it.

### Patients’ uptake of lifestyle-related recommendations

Eighty-one percent of patients responded that they exercised, 73% that they maintained a reasonable weight, 94% that they ate a healthy diet, 91% that they avoided smoking, and 97% that they limited their alcohol intake. Of the 56 women who reported having had children since receiving their genetic testing result, 96% reported breastfeeding.

## Discussion

We found no significant changes in relation to use of germline *BRCA1/2* testing. The lack of significant changes may reflect the fact that guidelines were developed by consensus and reflected current accepted practice that was already in place when the clinical practice guidelines were codified. Furthermore, post-guideline reasons for testing were provided for all tests not meeting guideline-based testing criteria. These findings suggest that current guideline-based testing criteria are too narrow, and that in practice FCCs apply broader criteria when offering testing, taking into account family-specific and individual factors. If these are considered reasonable factors to guide testing, then consideration should be given to amending guidelines to ensure equity of access to testing across the country.

Regarding rates of documentation of risk management recommendations in files of carriers, rates increased significantly from pre- to post-guideline for 6/9 risk management strategies, and these six included the most clinically effective strategies, risk-reducing mastectomy and salpingo-oophorectomy. No statistically significant increases in documentation were observed for recommendation of: risk-reducing salpingo-oophorectomy for breast cancer prevention; screening from 5 years prior to earliest age affected if breast cancer has been diagnosed in this family < 35 years; and annual breast cancer screening in carriers from > 50 years. However, regarding the guidelines on screening from > 50 years, 50 and 67% of pre-and post-guideline files respectively stated the patient was young, and that it would be discussed at an appropriate age. Regarding screening during pregnancy, 62 and 67% of pre-and post-guideline files respectively stated that management of screening during pregnancy was left to the specialist.

This study shows that the three risk management strategies with the highest rates of documentation of advice in files post-release of guidelines (risk-reducing salpingo-oophorectomy for ovarian cancer prevention, 98%; risk-reducing bilateral mastectomy, 92%; and breast cancer screening in 30–50 year old women, 88%) are risk management strategies with a strong evidence base. These results show that FCC staff prioritise advice on strategies with proven efficacy. This study also found low rates of post-guideline documentation of annual breast cancer screening recommended for women aged > 50 years (49%); amongst women interviewed aged > 50 years, only 30% women had received advice on annual mammography. These low rates of documentation of advice may be because 92% of women aged > 50 years were already having regular breast screening based on their family history prior to testing. Further, it is possible that more carriers were provided with advice on annual breast screening for women > 50 years than was documented in their clinic files; however, without documentation, carriers and their doctors have no opportunity to refer to it for advice. Ensuring that women aged > 50 years receive a recommendation for annual breast screening consistently, along with improved documentation, may result in even higher percentages of carriers taking up annual breast screening. It is important to highlight that we have not stratified documentation rates with the age of carriers at the time of delivery of the advice. Clearly advice, and therefore documentation, may have been tailored to the specific individual learning their mutation status and certain risk management strategies omitted from the discussion if they were not age- or life-stage-appropriate.

The significant increases in concordances pre- to post-guideline release for the majority of risk management strategies is in contrast to our findings on concordance with regard to guidelines on advice related to Lynch syndrome, where we found statistically significant increases in concordance for only two out of eight risk management strategies [20]. It might be speculated that for Lynch syndrome FCC clinicians are more likely to rely on other non-genetics specialists to provide risk management advice and management, who may not be as familiar with the national eviQ guidelines. It is possible that there are more non-genetics specialists outside FCCs with the required expertise to oversee risk management for carriers of *BRCA1/2* pathogenic variants, and/or that FCC staff are reluctant to leave direction for their risk management to non-genetics specialists leading them to provide specific risk management advice more readily compared to that for Lynch syndrome. The FCC clinicians may feel more confident in providing risk management advice to *BRCA1/2* pathogenic variant carriers compared to those with variants in MMR genes; if so, this may be the result of a greater volume of research being available regarding the efficacy of risk management in *BRCA1/2* pathogenic variant carriers and/or FCC staff being more experienced in providing advice to such carriers, given that most patients attending FCCs are from hereditary breast ovarian cancer families rather than families with Lynch syndrome [22].

When comparing the rates of documented advice and uptake amongst carriers of risk management strategies, fortunately, it was found that the rate of uptake of annual mammography amongst women aged > 50 years (92%) and uptake of no MRI or mammography in pregnant women (78%) was much higher than the advice documented in the clinic file. This suggests that once again either the advice was not documented in the clinic file, or that women received additional recommendations from other health professionals. We also found that only 58% of women reported having been told that serum CA125 and/or transvaginal ultrasound is not recommended, and this could have resulted in some women having ovarian cancer screening in the false belief it was beneficial. Fortunately 75% of women > 40 years had risk-reducing salpingo-oophorectomy at the time of the survey; nonetheless there is still room to improve the delivery and documentation of this important piece of advice. We did not ask detailed questions why 25% of women > 40 years had not had the surgery. While some are likely to be in their 40s and not at the right life stage to contemplate menopause or have other co-morbidities that render surgery inappropriate, there is still an important number who are eligible for surgery; strategies need to be developed to engage this group in order for them to gain the most clinical benefit from their genetic diagnosis [23].

We also found that only 6% of women who had not had a risk-reducing bilateral mastectomy were taking risk-reducing medication. This low rate of uptake of risk-reducing medications is consistent with literature showing that the actual reported uptake of tamoxifen by women at increased risk for breast cancer is low e.g. [24–26] However, the low uptake rate provides an interesting contrast with our findings on the uptake of aspirin in carriers of pathogenic variants in mismatch repair genes, which showed that 67% of carriers took aspirin for risk reduction [20]. The difference in findings may be related to aspirin having fewer perceived side-effects than tamoxifen and tamoxifen having implications for reproduction in women. In support of this explanation, our study showed that as many as 56% of women agreed “very much” that the side-effects of these medicines may affect their decision whether or not to take them. The similar side-effects (infertility and menopause) also prevents some women having risk-reducing salpingo-oophorectomy at an appropriate age [27]. Clearly more research to address and manage these fears and the side-effects are essential, if the uptake of risk-reducing medication and salpingo-oophorectomy is to be optimised.

The study’s strengths and limitations should be noted. Strengths included the fact that almost all Australian FCCs participated, leading to low institutional bias and high representativeness and generalisability of the data, providing nationwide data on the impact of guidelines relating to carriers of pathogenic variants in *BRCA1/2*. Inter-rater reliabilities of assessments were excellent. Limitations of the study are a relatively low participation rate of carriers in the interview study (52%); due to ethical stipulations we were not able to follow up non-responders more than once. However, similar participation rates have been reported by other studies using medical records [28, 29]. Another limitation is that no data were collected on sociodemographic variables (with the exception of age) to avoid participant fatigue, limit the time taken to interview patients, and to comply with the low-risk ethics approval status of the study. In this study, we only measured carriers’ compliance with risk management guidelines; qualitative and quantitative studies are required to assess the specific barriers to adoption of guidelines in order to provide the basis for the development of tailored interventions to address specific barriers. Finally, the current study did not assess how carriers were counselled with respect to risk management strategies, for example, the absolute risk of breast and/or ovarian cancer related to pathogenic variants in *BRCA1/2*, morbidity of risk-reducing surgery, and the effectiveness of cancer surveillance. The way carriers are counselled is likely to impact on whether they have made an informed decision. Future studies should assess information-giving and counselling behaviours during counselling sessions, for example by digitally recording consultations and subjecting recordings to a rigorous communication analysis [30].

## Supplementary Information

**Additional file 1.**

**Additional file 2.**

**Additional file 3.**

## Data Availability

The datasets used and/or analysed during the current study are available from the corresponding author on reasonable request.
