# Supplementary material for: Impact of national guidelines on use of BRCA1/2 germline testing, risk management advice given to women with pathogenic BRCA1/2 variants and uptake of advice
Source: Hered Cancer Clin Pract. 2021 Apr 9;19:24. doi: 10.1186/s13053-021-00180-3 (PMC8035714; doi:10.1186/s13053-021-00180-3)
Supplement: Supplementary file 1 — Additional file 1. [file 13053_2021_180_MOESM1_ESM.docx]

**File Audit Checklist – Genetic Testing for BRCA1/2**

1. PATIENT DETAILS:
2. Patient ID: _____________
3. Clinic: _________________ b. Date of genetic test (dd/mm/yyyy): _____/_____/________
4. REFERAL GUIDELINES:
   1. Is the mutation prediction score for this patient higher than at least one of the following? **Yes**

| Manchester score: ______. Is this ≥16? |  |  |  |
| --- | --- | --- | --- |
| BOADICEA: ______. Is this ≥ 10%? |  |  |  |
| BRCAPRO: ______. Is this ≥ 10%? |  |  |  |
| ❑ A score has not been indicated in the file |  |  |  |
| b. | Does the patient have triple negative breast cancer ≤ age 40 years? | |  |
| c. | Does the patient have an isolated high grade (Grades 2 & 3) invasive non-mucinous ovarian, fallopian tube or primary peritoneal cancer < age 70 yrs? | |  |
| d. | Does the patient have an invasive non-mucinous ovarian, fallopian tube or primary peritoneal cancer and a family history (defined as having one of the following:  ❑ a first-degree relative diagnosed with breast cancer at an age younger than 60 years;  ❑ a first-degree relative diagnosed with ovarian cancer at any age;  ❑ a combination of two of more first-degree relatives with breast or ovarian cancer; or  ❑ a male first-degree relative diagnosed with breast cancer at any age.)? | |  |
| e. | Is the patient from a population where a common founder mutation exists and has a personal and/or family history (defined as having one of the following:  ❑ a first-degree relative diagnosed with breast cancer at an age younger than 60 years;  ❑ a first-degree relative diagnosed with ovarian cancer at any age;  ❑ a combination of two of more first-degree relatives with breast or ovarian cancer; or  ❑ a male first-degree relative diagnosed with breast cancer at any age.)? | |  |

If there is a ‘✓’ in the yes column, then testing is compliant to eviQ

**❑_1_ Compliant ❑_0_ Non-compliant**

1. If non-compliant, were any reasons given for non-compliance (if reason is not clearly stated in the letters or contact notes, discuss this with a GC representative from the FCC)?

❑_0_ No

❑_1_ Yes, enrolled in the Treatment Focused Genetic Testing (TFGT) study.

❑_2_ Yes, gender imbalance – pedigree includes mostly male.

❑_3_ Yes, small family size – not enough people in the pedigree to allows an estimation of prevalence.

❑_4_ Yes, there is ovarian cancer in the family.

❑_5_ Yes, patient was adopted.

❑_6_ Yes, the patient did not know about their family history.

❑_7_ Yes, patient insisted on having a genetic test. If yes, it was: ❑ self-funded / ❑ publicly funded

❑ Yes, other: __________________________________________________________________

1. Calculate the Manchester and BRCAPRO scores (instructions overleaf): _______

**Audit completed by (initials): ____________ Date of audit (dd/mm/yy): ___/____/_____**

**CALCUATING A MANCHESTER SCORE:**

From: <https://www.eviq.org.au/Protocol/tabid/66/id/1143/Default.aspx?popup=1>

| **Cancer and patient age** | **Combined BRCA1/BRCA2** |  | **Pathology adjustments of breast cancer score in index case  (at any age)** | |
| --- | --- | --- | --- | --- |
| FBC <30 | 11 |  | Her2+ve* | -4 |
| FBC 30 - 39 | 8 |  | Lobular | -2 |
| FBC 40 - 49 | 6 |  | DCIS only (no invasive cancer) | -1 |
| FBC 50 - 59 | 4 |  | LCIS only* (no invasive cancer) | -4 |
| FBC>59 | 2 |  | Grade 1 IDC | -2 |
| MBC<60 | 13 |  | Grade 2 IDC | 0 |
| MBC>59 | 10 |  | Grade 3 IDC | +2 |
| Ovarian cancer<60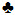 | 13 |  | ER+ve | -1 |
| Ovarian cancer>59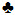 | 10 |  | ER-ve | +1 |
| Pancreatic cancer | 1 |  | Grade 3, triple -ve* | +4 |
| Prostate cancer<60 | 2 |  |  |  |
| Prostate cancer>59 | 1 |  |  |  |

| **KEY:** | | |
| --- | --- | --- |
| FBC: female breast cancer | MBC: male breast cancer | DCIS: ductal carcinoma in situ |
| LCIS: lobular carcinoma in situ | IDC: infiltrating ductal carcinoma | ER: Oestrogen receptor |
| Triple –ve: oestrogen, progesterone and Her2 receptor –ve | | |
| *These adjustments are final, and no further adjustment based on other pathological features is necessary | | |
| 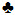Only epithelial (endometrioid, serous, clear cell, not otherwise specified) and granulosa cell pathology to be counted. No score is given for mucinous, borderline or germcell ovarian tumours | | |

| **RULES:** |
| --- |
| - score from the person you are potentially testing (index case) |
| - scores are added for each cancer in a direct blood lineage (one side of family only). No consideration is given for prior testing in these combined scores. |
| - in bilateral breast cancer, each breast cancer should be scored separately |
| - cancer through two unaffected females > 60 years is discounted |
| - DCIS is included |
| - a combined score of 16 points can be used as a 10% threshold in non-founder populations |

References

1. [Evans, D. G., F. Lalloo, A. Cramer, et al. 2009. "Addition of pathology and biomarker information significantly improves the performance of the Manchester scoring system for BRCA1 and BRCA2 testing." J Med Genet 46(12):811-817.](https://www.eviq.org.au/Reference/tabid/67/id/3165/Default.aspx)
