# Supplementary material for: Impact of national guidelines on use of BRCA1/2 germline testing, risk management advice given to women with pathogenic BRCA1/2 variants and uptake of advice
Source: Hered Cancer Clin Pract. 2021 Apr 9;19:24. doi: 10.1186/s13053-021-00180-3 (PMC8035714; doi:10.1186/s13053-021-00180-3)
Supplement: Supplementary file 2 — Additional file 2. [file 13053_2021_180_MOESM2_ESM.docx]

**File Audit Checklist – Risk Management for unaffected female BRCA1 gene mutation carriers**

1. PATIENT DETAILS:
2. Patient ID: _____________
3. Clinic: _________________ d. Date genetic test returned (dd/mm/yyyy): _____/_____/________
4. RISK MANAGMENT GUIDELINES

[**Abbreviations**: RRSO - Risk-reducing salpingo-oophorectomy, US – ultrasound, CBE – clinical breast examination, MMG mammogram (digital if available), MRI – magnetic resonance imaging.]

Have the following been raised as potential risk management strategies:

| **BREAST CANCER**  Surgical | | **Yes** | **No** | **If no, reason for non-compliance:** |  | **Compliant** |
| --- | --- | --- | --- | --- | --- | --- |
| a. | Bilateral risk-reducing mastectomy:  no nipple sparing surgery, no surveillance?  nipple sparing surgery, surveillance? |  |  |  |  |  |
| b. | Has RRSO been discussed, ≤40 yrs or while premenopausal? |  |  |  |  |  |
| Surveillance | |  |  |  |  |  |
| c. | If breast cancer has been diagnosed in this family <30 years, has screening been recommended from 5 yrs prior to earliest age affected? |  |  |  |  |  |
| d. | If not (c), has screening been suggested from age 30 years as below:  ❑ 30 – 50 years – annual MRI+MMG (if MRI unavailable, annual MMG+US), consider 6 month interval US+CBE  ❑ >50 years – annual MMG +/- US + CBE  ❑ if pregnant - no MRI or MMG, consider US |  |  |  |  |  |
| Risk-reducing medication | |  |  |  |  |  |
| e. | Risk-reducing medication recommended? |  |  |  |  |  |

**OVARIAN/ FALLOPIAN TUBE CANCER**

| Surgical | |  |  |  |  |  |
| --- | --- | --- | --- | --- | --- | --- |
| f. | RRSO from age 35 years in mutation carriers? |  |  |  |  |  |
| Surveillance | |  |  |  |  |  |
| g. | If breast cancer has been diagnosed 30-35 years, CA125 and/or transvaginal ultrasound (TVU) considered in those with a strong family history of ovarian cancer and have not finished their family or do not wish to undergo RRSO? |  |  |  |  |  |
| h. | If not g), has the patient been told that serum CA125 and/or transvaginal ultrasound (TVU) is **not** recommended? |  |  |  |  |  |
| j. | Considered fallopian tube ligation or other means for contraception? |  |  |  |  |  |

**ALL CANCERS**

| i. | Have the following been suggested to the patient to:  ❑ exercise,  ❑ maintain a reasonable weight,  ❑ breast feed,  ❑ avoid prolonged use of oral contraceptive pill  ❑ avoid post-menopausal HRT |  |  |  |  |  |
| --- | --- | --- | --- | --- | --- | --- |

Calculate a compliance score (every ‘✓’ = 1 point): ______ /6

**Audit completed by (initials): ____________ Date of audit (dd/mm/yy): ___/____/_____**
