# Supplementary material for: Impact of national guidelines on use of BRCA1/2 germline testing, risk management advice given to women with pathogenic BRCA1/2 variants and uptake of advice
Source: Hered Cancer Clin Pract. 2021 Apr 9;19:24. doi: 10.1186/s13053-021-00180-3 (PMC8035714; doi:10.1186/s13053-021-00180-3)
Supplement: Supplementary file 3 — Additional file 3. [file 13053_2021_180_MOESM3_ESM.docx]

**Checklist – Risk management for unaffected female BRCA1 gene mutation carriers**

1. PATIENT DETAILS:
2. Patient ID: _____________
3. Clinic: _________________
4. RISK MANAGMENT GUIDELINES

[**Abbreviations**: RRSO - Risk-reducing salpingo-oophorectomy - ovaries and fallopian tubes removed;

US – ultrasound - a small handheld transducer is passed over the breast;

CBE – clinical breast examination - clinician checks the breasts;

MMG mammogram (digital if available) - the breasts are compressed between two flat plates while an x-ray is being taken;

MRI – magnetic resonance imaging - lying face down on a table and being moved into a large tube-shaped machine]

| **Surgical** | | **Yes** | **No** | **If no, can you explain why you have made this decision at this time** | | | | |
| --- | --- | --- | --- | --- | --- | --- | --- | --- |
| a. | Have you had both of your breasts removed (double mastectomy)? |  |  |  | | | | |
| b. | *If yes,* Do you check around the breast reconstructions or chest wall if you have not had a reconstruction? |  |  |  | | | | |
| c. | Have you had your ovaries and fallopian tubes removed (a risk-reducing salpingo-oophorectomy [RRSO])? |  |  |  | | | | |
| **Screening** | |  |  |  | | | | |
| d. | *If you still have your breast(s) and/or ovaries ->* Are you having:  *If 30 – 50 years –>* ❑ annual MRI+MMG (+/- US)?  *If >50 years –>* ❑ annual MMG +/- US + CBE?  *If pregnant and 30 yrs or over ->* ❑ no MRI or MMG, consider US? |  |  |  | | | | |
| e. | Have you been told that ovarian cancer screening with blood tests ( CA125) and/or an ultrasound (TVU) is **not** recommended because it is ineffective? |  |  |  | | | | |
| **Risk-reducing medication** | |  |  |  | | | | |
| f. | Are you taking a medicine to reduce your risk of developing breast cancer?  If yes, which one?  ❑ tamoxifen  ❑ raloxifene  ❑ exemestane |  |  |  | | | | |
| *To what extent do you agree that each of the following influences your decision whether or not to use a medicine to reduce your risk of developing breast cancer?* | | **Very much agree** | | | **Somewhat agree** | | **Somewhat disagree** | **Very much disagree** |
| g. | The side-effects of these medicines. |  | | |  | |  |  |
| h. | Other people’s experiences with it. |  | | |  | |  |  |
| i. | That it is also used as a cancer medicine. |  | | |  | |  |  |
| j. | It is a reminder of your breast cancer risk. |  | | |  | |  |  |
| **General** | | **Yes** | **No** | **If no, can you explain why you have made this decision at this time** | | | | |
| k. | Do you:  ❑ exercise,  ❑ maintain a reasonable weight,  ❑ have a healthy diet,  ❑ breast feed,  ❑ avoid smoking, and  ❑ limit alcohol intake. |  |  | | |  | | |

**Completed by (initials): ____________ Date of interview (dd/mm/yy): ___/____/_____**
